# Special Issue: “A Wider Look at Pediatric Primary Headaches”

**DOI:** 10.3390/life14101341

**Published:** 2024-10-21

**Authors:** Vincenzo Raieli, Vittorio Sciruicchio

**Affiliations:** 1 Child Neuropsychiatry Unit-ISMEP-ARNAS CIVICO, 90100 Palermo, Italy; 2 Children Epilepsy and EEG Center, San Paolo Hospital, ASL Bari, 70132 Bari, Italy; vittorio.sciruicchio@asl.bari.it

Headaches represent a common and debilitating neurological disorder among the pediatric population. Despite the significant amount of data on pediatric headaches, they continue to present a major challenge for clinicians. Headaches are likely the most frequent neurological symptom for which children and adolescents are referred to a doctor or an emergency department [1].

It is important to recognize that children are not simply “little adults”. Pediatric primary headaches can vary significantly throughout childhood and adolescence, not only in their clinical and prognostic features but also in their response to treatment. Aside from migraines, other pediatric headaches—such as tension headaches or primary stabbing headaches—are also quite common. In contrast, conditions such as TACs (trigeminal autonomic cephalalgias) are more rarely studied and are not as well understood. Therefore, more information is needed regarding the unique clinical features of these headaches, especially in early childhood, along with the adaptability of the latest IHS diagnostic criteria and the long-term outcomes as children age. Additionally, it would be of great value to investigate possible changes in headache characteristics in the COVID-19 era and responses to pharmacological and non-pharmacological therapies.

Our main aim for this Special Issue was to collect clinical observations and experimental evidence that highlighted the unique aspects of other primary pediatric headaches in the early stage of life. We hope that this collection will assist fellow clinicians in effectively recognizing and treating these conditions in younger patients.

Correnti et al. [2] present a significant number of pediatric clinical cases involving orofacial pain not secondary to orofacial disorders. While these cases may exhibit characteristics of primary headaches, their atypical location can lead to misdiagnosis, resulting in unnecessary diagnostic tests or invasive therapies. The authors also describe less well-known syndromes, such as trochlear migraine and red ear syndrome.

Kalika and Monteith [3] provide an in-depth review of new persistent daily headache (NPDH), a primary headache disorder characterized by the sudden onset of continuous pain that is resistant to treatment. Despite its prevalence in the pediatric population, NPDH remains poorly studied and underdiagnosed. The authors offer an up-to-date overview of the clinical history, pathophysiology, and treatment options of this complex and challenging syndrome.

Tozzi et al. [4] have published a systematic review on benign paroxysmal torticollis (BPT) and its association with migraines. BPT, often misunderstood, can cause significant alarm, as it primarily affects newborns and young children. This review highlights data on the relationship between BPT and various forms of migraine.

The Padua group [5,6] offer two systematic reviews addressing primary stabbing headache [5]—a condition that is less rare than is often believed—and cold stimulus headache [6], which is much less frequently described. Primary stabbing headache, included among indomethacin-sensitive headaches, can be alarming, and its connection to other primary headaches is also explored. The researchers [6] further document the first case of cold headache triggered by low temperatures rather than by the ingestion of cold substances.

Lastly, Baglioni et al. [7] present a detailed narrative review of tension-type headache (TTH) in the pediatric population. TTH is the most common form of primary headache, yet it remains underdiagnosed and poorly defined, often overlapping with migraines during childhood and adolescence. This review outlines the diagnostic process for TTH in pediatric patients, potential outcomes during developmental years, and appropriate therapeutic strategies. TTH presents a significant challenge for pediatric specialists.

In conclusion, this Special Issue focuses on non-migraine primary headaches in childhood, some quite common, others rarer, and emphasizes the need for further studies to improve the diagnostic process and treatments.
